# Supplementary material for: Time‑resolved multi-omic analysis of paclitaxel exposure in human iPSC‑derived sensory neurons unveils mechanisms of chemotherapy‑induced peripheral neuropathy
Source: Cell Death Dis. 2026 Feb 10;17(1):211. doi: 10.1038/s41419-026-08445-2 (PMC12921266; doi:10.1038/s41419-026-08445-2)
Supplement: Supplementary file 15 — Dataset 1. [file 41419_2026_8445_MOESM15_ESM.zip › CDD_Open_Data/04_RNA_Seq/Timeline_panel_plots_etc_2023-01-23_1609.zip!/Panel_Plots_etc/Evidence_Plots/72h/evidence_plot_standard contrasts_msigdb_reactome_72h_PTX_vs_DMSO_ID0_M9648_REACTOME_FORMATION_OF_TUBULIN_FOLDING_INTERMEDIATES_BY_CCT_TRIC.pdf]

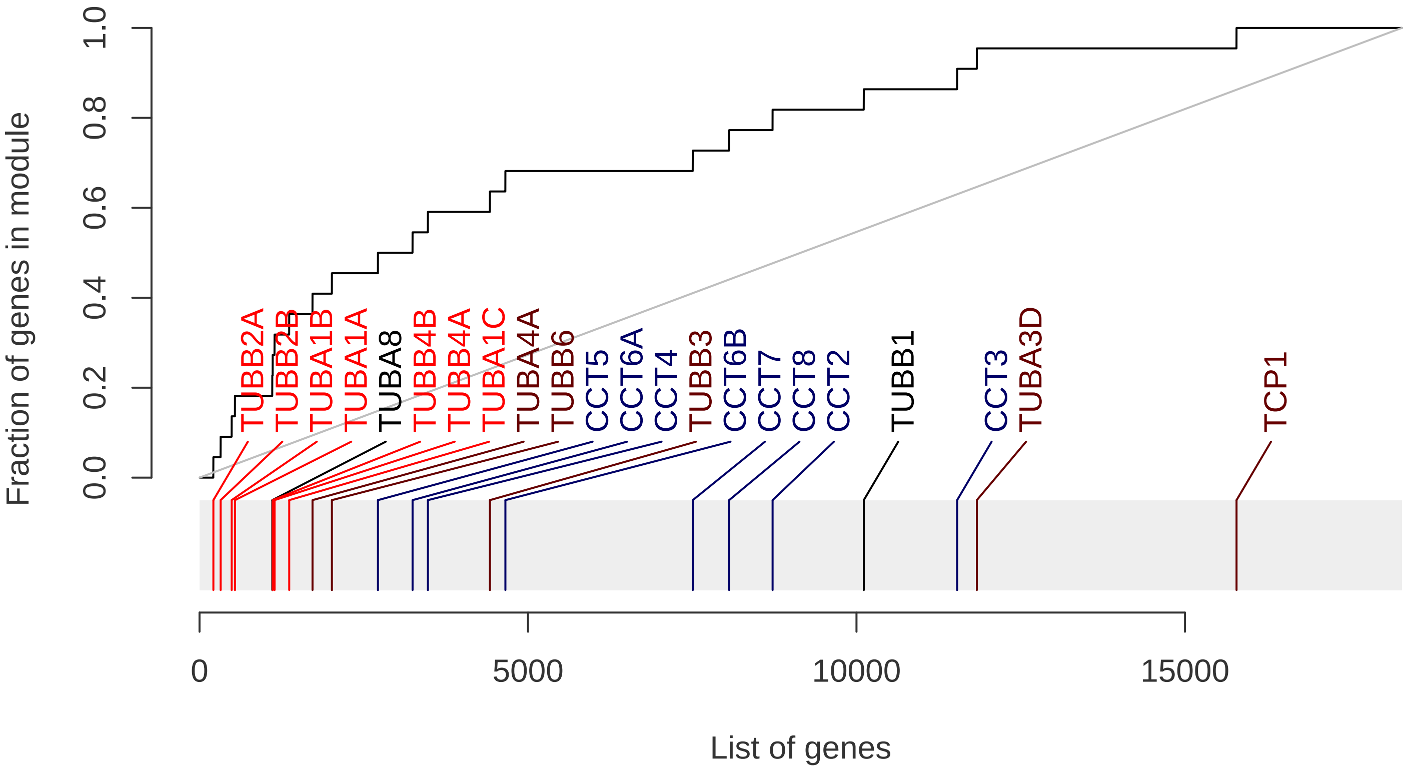

Fraction of genes in module
0.0
0.2
0.4
0.6
0.8
1.0
TUBB2A
TUBB2B
TUBA1B
TUBA1A
TUBA8
TUBB4B
TUBB4A
TUBA1C
TUBA4A
TUBB6
CCT5
CCT6A
CCT4
TUBB3
CCT6B
CCT7
CCT8
CCT2
TUBB1
CCT3
TUBA3D
TCP1
0
5000
10000
15000
List of genes
